# Residence in segregated settlements (colonies) rather than Roma identity increases the risk of unfavourable mental health in Hungarian adults

**DOI:** 10.3389/fpubh.2023.1205504

**Published:** 2023-08-01

**Authors:** Barnabás Oláh, Éva Bíró, Karolina Kósa

**Affiliations:** ^1^Department of Behavioural Sciences, Faculty of Medicine, University of Debrecen, Debrecen, Hungary; ^2^Department of Public Health and Epidemiology, Faculty of Medicine, University of Debrecen, Debrecen, Hungary

**Keywords:** Hungarian, Roma, segregated settlements, mental health, life satisfaction, subjective well-being, social support

## Abstract

**Background:**

Roma are the largest and most disadvantaged minority in Europe, but there is few research on how mental health and social support of Roma people living in segregated settlements compares to the majority population. Our aim was to compare the subjective well-being, life satisfaction, mental status, and social support of representative samples of adults living in segregated settlements (colonies) and identifying as Roma with those of the general population in Hungary.

**Methods:**

A cross-sectional study was conducted with random samples of 417 individuals from the general Hungarian adult population (55.6% female, mean age = 43.89 ± 12.61 years) and 394 adults living in segregated settlements (colonies) (73.9% female, mean age = 42.37 ± 12.39 years). Demographic questions were used as well as the WHO Well-Being Index (WBI-5), the single item Life Satisfaction Scale, the 12-item version of the General Health Questionnaire (GHQ-12), and the Oslo Social Support Scale (OSSS-3).

**Results:**

Residents of colonies reported significantly lower levels of subjective well-being and life satisfaction than the general population. The proportion of individuals at high risk for mental morbidity was more than twice as high among colony dwellers (16.4%) as in non-colony dwellers (7.6%). Similar unfavorable differences were seen at the expense of self-identified Roma compared to self-identified Hungarians but no difference was found in terms of social support either by type of residence or ethnicity. 32.2% of colony-dwellers self-identified themselves as Hungarian. Mental health assessed by principal component was directly determined by settlement type of permanent residence, age, educational attainment, employment, financial status, and social support but not ethnic identity.

**Conclusion:**

The study based on representative data shows that residents of segregated settlements are in worse mental health than those not living in colonies; that housing segregation is not limited to Roma people, and that housing conditions and financial status are major social determinants of mental health for which data must be collected to avoid using self-reported Roma identity as a proxy measure of socioeconomic deprivation.

## 1. Introduction

Roma people constitute the largest minority in the European Union (EU) and in Hungary, estimated to be 3.7–8.8% of the country’s total population (1). Many of them suffer from economic and social deprivation compounded by anti-Gypsyism, prejudice and discrimination in spite of continued efforts to improve their situation. One of the features of deprivation has been that many Roma live in segregated colonies (2, 3). Compared with the non-Roma population in Europe, Roma tend to be in less favorable health, reflected by lower life expectancy, higher infant mortality, and lower vaccination rates (4). Compared with the non-Roma population, they are at increased risk for diseases and chronic illnesses (5–7).

A comparative study of representative samples of the Hungarian Roma and non-Roma population found that the subjective health of Roma adults living in colonies was similar to that of non-Roma people in the lowest socioeconomic quartile of the majority population and socio-economic differences explained worse health status though not health behavior (8).

Unfavorable health among Roma has been shown to be associated with social factors including limited education, substandard living conditions, sustained poverty as well as inadequate access to health services (9). Substandard living conditions do not only mean that many Roma live in poor quality houses but also in many cases these houses are located in segregated parts of settlements (colonies) without public amenities in member states of the EU (4) and in Hungary (10). These segregated settlements are aggregations of substandard houses located usually on the fringes of cities and villages. By definition, colonies have a minimum of four dwelling units that are distinguishable by lower quality, higher population density, and sub-par environmental conditions compared to other dwelling units within the same or neighboring settlement (if the colony is outside of a settlement) (10).

Unfavorable or below-average social determinants of health contribute to chronic stress and are associated with increased risk of many common mental disorders (11), therefore Roma should be considered at high risk not only for somatic but also for psychological morbidity. Persons from Roma communities are several times more likely to experience anxiety, depression, suicidal thoughts and attempts (12, 13) and problems (14). Roma in a nonrepresentative sample of 8,399 Roma and 3,598 non-Roma persons from Central and Southeastern Europe had lower well-being compared with non-Roma entirely due to poorer health, lower income, lower education, poorer housing quality, shame about Roma identity, and higher perceived discrimination (15). The risk of severe depression assessed by the eight-item Patient Health Questionnaire (PHQ-8) was significantly, more than twice as high, and the mean of life satisfaction measured by a single item was significantly lower among Hungarian Roma adults than in the general population in the 2014 European Health Survey of the country (16). Roma school children in Romania and Bulgaria were found to have a more than twofold odds of any internalizing or externalizing disorder compared to their non-Roma peers (17).

While data have been available about the health status of Roma persons in general, there has been a dearth of studies on mental health and its determinants among Roma living in segregated settlements. This is due to a host of factors such as lack of reliable data on segregated settlements, difficulties of access, mistrust of outsiders, problems of digital literacy that requires paper-based data collection (18). As a result, remarkably little research has been conducted on how the mental health of the segregated population living in Roma settlements compares to the majority population in the countries where they live. To our knowledge, there is no study that compares the mental health of representative samples of adult residents of Roma settlements with the general adult population, taking into account not only the negative aspects such as the risk of mental disorders but also the positive aspects of mental health, that is, subjective well-being and life satisfaction.

Our aim was to compare mental health characteristics assessed by different variables in adult inhabitants of segregated settlements (colonies) and that of the non-colony dwelling adult population. We also wanted to investigate mental health characteristics in our samples by self-reported ethnicity regardless of type of residence. We used quantitative measures to assess negative aspects of mental health, the risk for mental morbidity, and positive aspects of mental health beyond the absence of mental illness such as subjective well-being and its cognitive component, life satisfaction. We hypothesized that the mental health of members of Roma minority groups would be less favorable than that of the majority, therefore we also aimed at investigating to what extent this may be due to living in segregated settlement, minority identity, and differences in the major social determinants of health such as education, financial and economic status, or social ties.

Supportive social relations have been identified in the last half century as mechanisms by which interpersonal relationships have protective effects against the impact of stress and may reduce the risk of psychopathology (19). Social ties are of major importance for Roma since they belong to a collectivist culture as has been shown in Sweden (20) and Slovakia where social support from family and friends decreased the risk of not being able to access health care (21).

Given the importance of social support in maintaining positive mental health, preventing mental disorders, and accessing health services, we aimed at investigating the differences in perceived social support by type of permanent residence (colony dwellers yes or no) and by self-reported ethnicity (Roma vs. Hungarian). We hypothesized that lower social support among Roma account for poorer mental health. Social support may be operationalized in several ways; our study used three distinct indicators: marital status; subjective assessments of receiving support and help from family and neighbors; and availability of persons to discuss personal problems.

## 2. Methods

### 2.1. Participants and data collection

A complex cross-sectional health examination and behavior survey was carried out in 2018 that was described in detail elsewhere (22). Briefly, 18-to 64-year-old individuals living in 25 randomly selected segregated colonies were invited to participate to represent the adult Hungarian Roma population in two counties (Hajdú-Bihar and Szabolcs-Szatmár-Bereg) of Northeastern Hungary where Roma live in the highest numbers and where most segregated Roma colonies had been identified previously (10).

92 segregated colonies with more than 100 inhabitants previously identified in a nationwide survey (10) were included in the study. Of those, 25 colonies were randomly selected. Using household lists validated by GPs, 20 households were randomly selected in each colony, and one person aged 18 to 64 years from each household (N = 500) was invited to be interviewed in person at the respondent’s household by Roma university students under the supervision of public health coordinators. Of those invited, 404 persons agreed to participate.

The sample representing the general Hungarian adult population by age and gender consisted of randomly selected individuals aged 18 to 64 years living in private households in the same counties of Northeastern Hungary who were registered as clients of general practitioners in the General Practitioners Morbidity Sentinel Program (GPMSSP), a population-based registry established in 1998 to monitor the prevalence and incidence of chronic non-communicable diseases of major public health importance (23).

500–500 individuals were planned to be included in both samples of whom 404 colony dwellers and 417 persons from the general population agreed to participate. After removing 10 records with age above 64 years to keep the pre-defined age range, 394 records from colony dwellers and 417 from the general population were eligible for analysis.

All subjects gave informed consent for inclusion in the study prior to participation. The study was conducted in accordance with the Declaration of Helsinki, and the protocol was approved by the Ethics Committee of the Hungarian Scientific Council on Health (61327-2017/EKU).

### 2.2. Measures

#### 2.2.1. Demographic questionnaire

These items of the questionnaire-based health behavior survey were taken from the Hungarian version (24) of the European Health Interview Survey (EHIS) wave 2 (25). Data were collected on gender, date of birth (from which age in the year of the survey was calculated), marital status, number of persons in the household, educational attainment, economic activity, financial status. Type of residence (segregated settlement [colony] or not) was also registered. In order to simplify statistical evaluation and interpretation, we combined 11 categories of educational attainment into 4 (less than primary, primary, secondary, tertiary); 13 categories of economic activity into 3 (1: active: full time worker, part time worker, helper in family-owned business, full-time entrepreneur, part-time entrepreneur, casual employee or day laborer; 2: inactive because retired, disabled, unable to work, full-time student, cares for child, housewife or of other reason; and 3: unemployed). Since most but not all persons living in segregated settlements are Roma, self-reported ethnicity was also inquired about by one item taken from the questionnaire of the national census that asked about the primary identity of the respondent by choosing Hungarian or one of the 19 recognized ethnic groups, including Roma.

#### 2.2.2. Assessment of mental health status

##### 2.2.2.1. Well-being index of the world health organization

The WBI consists of five simple 5-point Likert questions that measure the respondent’s current psychological well-being over the past 14 days. Responses range from 0 to 5, where 5 means “almost always true” and 0 means “not true at all.” A total score is calculated by summing up all responses. The scale has high validity and reliability and has been used in many studies (26). Cronbach’s alpha for the scale was 0.86 in the present study.

##### 2.2.2.2. Single-Item Life Satisfaction Scale (LS)

Participants were asked to rate their life satisfaction on a scale of 1–10 as follows: Overall, how satisfied are you with your life today? where 10 represents “completely satisfied” and 1 represents “not at all satisfied.” The measure reflects the cognitive assessment of satisfaction with one’s life (27). Psychometric studies on large samples have shown that single-item measures of life satisfaction yield comparable results to multiple-item measures of the construct (28).

##### 2.2.2.3. General Health Questionnaire (GHQ)

The 12-item version (GHQ-12) is a screening instrument for psychological morbidity (having pathological levels of distress) in the general population and in community-based or non-psychiatric clinical settings. Respondents answer questions on a 4-point Likert scale. Cases are identified by rating symptoms as present or absent using the (0–0–1-1) method (29). The total score ranges from 0–12; scores above 4 reflect increased risk for psychological morbidity. Cronbach’s alpha was 0.89 in the present survey for this scale.

#### 2.2.3. Indicators of social support

##### 2.2.3.1. Marital status

The 5 categories of marital status were contracted into 2 categories (lives in marriage or registered cohabitation; unmarried-widowed-divorced).

##### 2.2.3.2. Oslo Social Support Scale (OSSS)

The OSSS consists of three items assessing the level of perceived social support. Perceived social support is based on the individual’s interpretation and subjective assessment of the support they believe is available for them. This instrument has been recommended for epidemiological and population-based surveys (30). Responses to the first question ranged from 1 to 4 and responses to the other two questions ranged from 1 to 5. Items 2 and 3 were reversed before adding up the scores to obtain a total score ranging between 3 to 14, higher scores indicating higher levels of social support. The Cronbach’s alpha reliability index of the questionnaire was 0.54 in our samples (general population: a = 0.59; colony dwellers: a = 0.48). We also calculated McDonalds omega coefficient to observe whether it yields higher internal consistency values, however we got somewhat similar results to Cronbach’s alpha (general population: Ω = 0.60; colony dwellers: Ω = 0.53).

##### 2.2.3.3. Support in personal matters

One question inquired about the availability of a person with whom the respondent could discuss personal matters, answering yes or no.

### 2.3. Statistical analysis

Statistical analyses were performed using IBM SPSS Statistics ver. 23.0 (IBM, Armonk, NY, United States) and Stata IC/16.1 (Stata Corp LLC, College Station, TX, United States). First, the demographic characteristics of the samples were described with mean ± standard deviation or proportions for the full sample, and by residence and ethnicity. Mean and standard deviation scores for subjective well-being, life satisfaction, and social support, and frequency categories of GHQ risk levels were calculated. Group comparisons of categorical variables were performed using Pearson’s chi-square test. Variances of continuous variables were tested with Levene’s test, then *t*-test for independent samples or Welch’s d test in case of unequal variances was applied to assess group differences.

Sociodemographic determinants of mental health variables were examined by 2 methods. First, each variable was analysed separately, using generalized linear models with entry method (GHQ, WBI, LS). For *post hoc* analysis, the adjusted Wald test was used.

Second, one composite was created from all 3 variables as follows. Spearman correlation of the 3 variables of mental status (WBI-5, LS, GHQ-12) showed strongly significant correlation (*p* < 0.001 for all pairs) so principal component analysis (pca) was used for data reduction to find a component that could be used in subsequent modeling as an outcome variable. Before that, the interval scales of WBI-5 and LS were reversed so that higher scores reflected less favorable mental health (increasing scores of GHQ reflect more symptoms and higher risk anyway). Next, the interval scores of all 3 variables were standardized to eliminate differences in scaling and variance. Principal component analysis with the standardized variables revealed that the first and only principal component with an eigenvalue over 1 explained 74% of the total variance (Comp1, Table 1).

**Table 1:** Description of mental health variables and their principal components.

Summary statistics of mental variables in the pca					Principal components		
	Mean	SD	Min-Max	SMC*	Component	Eigenvalue	Proportion
WBI (reversed)	−0.0164	0.989	−1.692; 3.436	0.540	Comp1	2.238	0.746
LS (reversed)	−0.0122	1.000	−1.203; 3.495	0.505	Comp2	0.449	0.149
GHQ	−0.0160	0.972	−1.980; 5.260	0.407	Comp3	0.311	0.103

WBI: Well-Being Index.

LS: life satisfaction.

GHQ: General Health Questionnaire.

SMC: squared multiple correlations of variables with each other after principal component analysis (pca).

**Figure 1:**
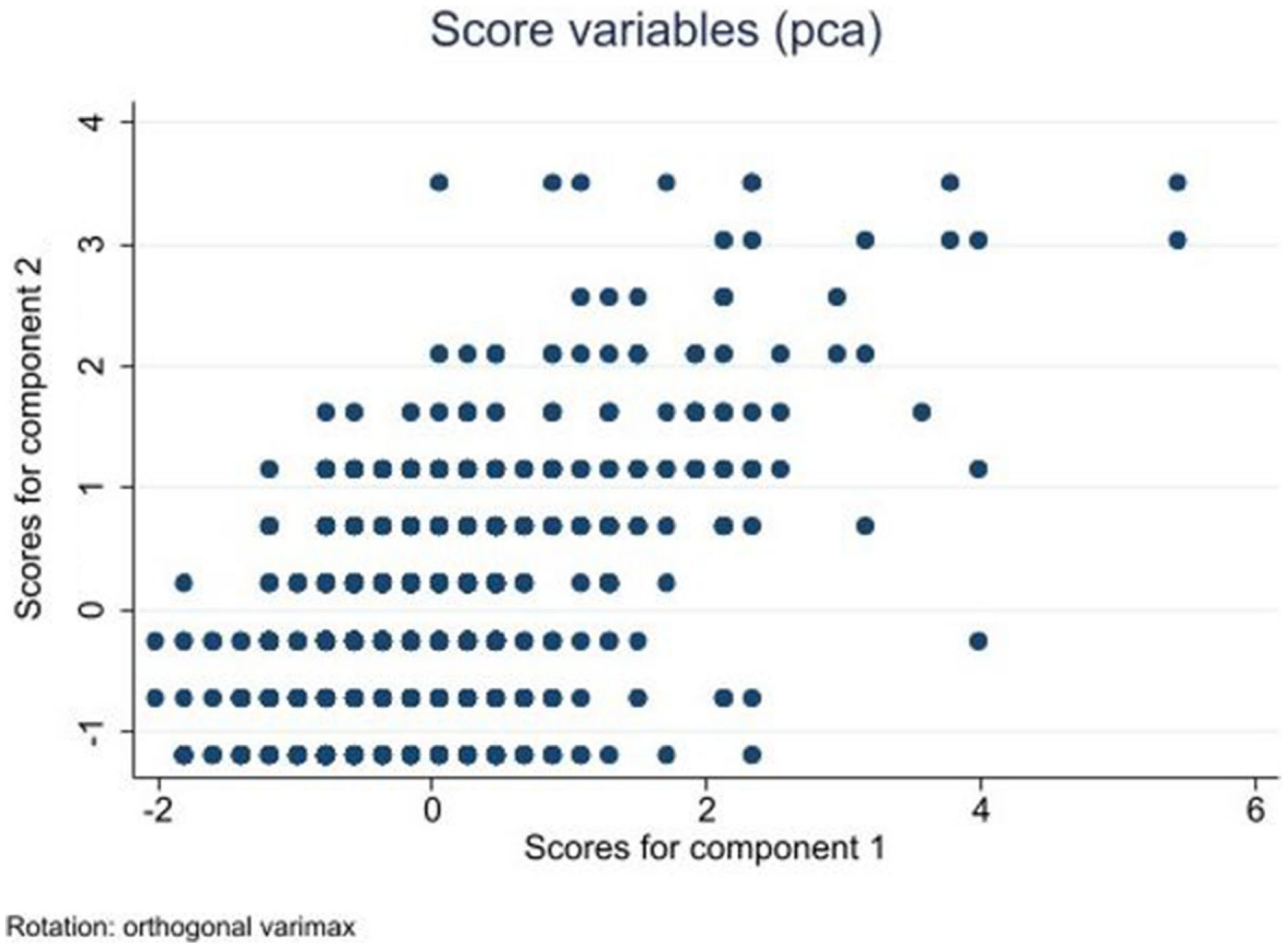
Scores for Components 1 and 2 after principal component analysis (pca) of mental health variables.

The Kaiser-Meyer-Olkin (KMO) measure of sampling adequacy was 0.701 supporting data reduction (31). Component1 was subsequently used as an outcome variable in model testing with higher values reflecting worse mental health (Figure 1).

Spearman correlation was used to identify independent variables significantly associated with Component1 that were the following: self-identified ethnicity, type of permanent residence (colony or not), age, educational attainment, economic activity, financial status, social support assessed by 3 items (OSSS) and a single item (support in private matters).

These 8 variables as determinants of mental health assessed by Component1 as dependent variable were examined by generalized linear modeling (glm) applying backward stepwise estimation that removed determinants at alpha = 0.1 from the model. Forward stepwise estimation in glm was used to investigate determinants of each mental health outcome separately. The adjusted Wald test was used for *post hoc* analysis. Alpha levels were set at 0.05.

## 3. Results

### 3.1. Demographic characteristics of the samples

67.8% of all colony dwellers identified themselves as Roma and 32.2% as Hungarian. In contrast, only 4.8% self-identified as Roma and 95.2% as Hungarian of those who lived in non-segregated settlements. Of all who identified as Roma, 93% lived in segregated settlements, and of all who identified as Hungarian, 24.1% lived in segregated conditions.

The sociodemographic characteristics were analysed by type of permanent residence (segregated or non-segregated settlements) and self-reported ethnicity (Table 2). There was no significant difference in terms of marital status between the subgroups but significant differences were seen in all other demographic variables. Those living in segregated vs. non-segregated settlements were no different in the distribution of age groups or mean age but self-identified Roma were significantly, 2.87 years younger compared to self-identified Hungarians. Those living in colonies and those who identified as Roma had significantly lower levels of education, significantly higher, at least twice as many were unemployed, at least twice as many of them were in bad or very bad financial situation, and the size of their household (persons living together) were also significantly bigger than that of those not living in colonies and not identifying as Roma.

**Table 2:** Demographic characteristics by type of residence and ethnicity.

Demographic variables^e^	Total	Type of permanent residence			Self-reported ethnicity		
		Segregated	Non-segregated	*p* value	Roma identity	Hungarian identity	*p* value
	(*n* = 811)	(*n* = 394)	(*n* = 417)		(*n* = 286)	(*n* = 523)	
Gender *n* (%)							
Male	288 (35.5)	103 (26.1)	185 (44.4)	<0.001^a^	82 (28.7)	206 (39.4)	0.003^a^
Female	523 (64.5)	291 (73.9)	232 (55.6)		204 (71.3)	317 (60.6)	
Age group *n* (%)							
18–29 y	149 (18.4)	80 (20.3)	69 (16.5)	0.380^a^	80 (20.3)	83 (15.9)	0.011^a^
30-44 y	267 (32.9)	128 (32.5)	139 (33.3)		100 (35.0)	167 (31.9)	
45–64 y	395 (48.7)	186 (47.2)	209 (50.1)		121 (42.31)	273 (52.2)	
Age mean ± SD	43.14 ± 12.51	42.36 ± 12.38	43.88 ± 12.61	0.084^b^	41.30 ± 12.55	44.17 ± 12.39	0.002
Marital status *n* (%)							
Married/ cohabiting	512 (63.7)	258 (66.3)	254 (61.2)	0.131^a^	180 (63.83)	309 (59.02)	0.169^a^
Other	292 (36.3)	131 (33.7)	164 (38.8)		103 (36.01)	213 (40.8)	
Unmarried	177 (22.0)	76 (19.5)	101 (24.3)		52 (18.18)	125 (23.72)	
Widowed	52 (6.5)	32 (8.2)	20 (4.8)		20 (6.99)	36 (6.83)	
Separated/ Divorced	63 (7.8)	23 (5.9)	40 (9.6)		31 (10.83)	56 (10.62)	
Educational attainment *n* (%)							
Less than primary	111 (13.8)	100 (25.7)	11 (2.7)	<0.001^a^	73 (25.9)	38 (7.3)	<0.001^a^
Primary	310 (36.8)	232 (59.6)	78 (18.8)		168 (59.6)	142 (27.2)	
Secondary	307 (38.2)	56 (14.4)	251 (60.5)		40 (14.2)	267 (51.2)	
Tertiary	76 (9.5)	1 (0.3)	75 (18.1)		1 (0.3)	75 (18.1)	
Economic activity *n* (%)							
Active	552 (69.2)	239 (61.8)	313 (76.2)	<0.001^a^	174 (62.6)	378 (72.7)	<0.001^a^
Inactive	173 (21.7)	89 (23.0)	84 (20.4)		60 (21.6)	113 (21.7)	
Unemployed	73 (9.1)	59 (15.2)	14 (3.4)		44 (15.8)	29 (5.6)	
Financial status *n* (%)							
very bad	31 (3.9)	24 (6.2)	7 (1.7)	<0.001^a^	18 (6.4)	13 (2.5)	<0.001^a^
bad	134 (16.8)	93 (23.9)	41 (10.1)		71 (25.2)	63 (12.2)	
fair	440 (55.2)	210 (54.0)	230 (56.4)		147 (14.5)	293 (56.9)	
good	167 (20.9)	57 (14.6)	110 (27.0)		41 (14.5)	126 (24.5)	
very good	25 (3.1)	5 (1.3)	20 (4.9)		5 (1.8)	20 (3.9)	
No. of persons in the household							
median (IQR^c^)	4 (2; 5)	5 (3; 6)	3 (2; 4)	<0.001^c^	5 (3; 6)	3 (2; 4)	<0.001^d^

a Pearson’s chi-squared test.

b Independent samples t test.

c interquartile range.

d Wilcoxon test.

e The categories do not add up to full sample size due to some missing data (excluding gender).

### 3.2. Mental health of the samples

Those living in non-segregated settlements and those identifying as Hungarians scored significantly, 1.75 and 1.29 points higher in subjective well-being (*p* < 0.001 for both) and life satisfaction (d = 0.57, *p* < 0.001, and d = 0.56, *p* < 0.001) compared to those living in colonies and identifying as Roma, respectively (Table 3). Regarding pathological distress measured by GHQ, the proportion of individuals at risk for psychological morbidity was more than twice as high among colony residents (16.4%) compared to those living elsewhere (7.6%, *p* < 0.001). By ethnicity, 1.7 times more Roma persons were at risk for psychological morbidity compared to Hungarians (17.8% vs. 10.3%, p < 0.001). No significant differences were found between the subgroups divided by residence or ethnicity regarding perceived social support assessed by the OSSS-3 scale or by support in personal matters assessed by one item (Table 3).

**Table 3:** Mental health characteristics and social support by type of residence and ethnicity.

Variables	Total	Type of permanent residence			Self-reported ethnicity		
		Segregated	Non-segregated	*p* value	Roma identity	Hungarian identity	*p* value
Subjective well-being, mean ± sd	16.75 ± 4.88	15.85 ± 5.26	17.6 ± 4.31	<0.001^b^	15.91 ± 5.17	17.2 ± 4.64	<0.001^b^
Life satisfaction, mean ± sd	7.44 ± 2.13	7.14 ± 1.81	7.71 ± 2.40	<0.001^b^	7.07 ± 2.46	7.63 ± 1.90	0.001^b^
Psychological distress *n* (%)^c^							
Low risk	704 (88.1)	327 (83.6)	377 (92.4)	<0.001^a^	235 (82.2)	469 (89.7)	<0.001^a^
High risk (GHQ-12 score > 4)	95 (11.9)	64 (16.4)	31 (7.6)		51 (17.8)	54 (10.3)	
Social support, mean ± sd	10.2 ± 1.99	10.09 ± 1.92	10.3 ± 2.05	0.139 ^b^	10.13 ± 1.97	10.22 ± 2.0	0.554
Support in personal matters (yes, %)	762 (95.4)	376 (96.7)	386 (94.1)	0.063	269 (95.4)	493 (95.4)	0.568

a Indicates the application of Pearson’s chi-squared test.

b Indicates the application of Welch’s d-test.

c The category does not add up to full sample size due to some missing data.

### 3.3. Modeling the individual variables of mental health

Pathological distress assessed by GHQ as scaled outcome variable, subjective well-being (WBI), and life satisfaction (LS) were each analysed separately by stepwise forward generalized linear modeling as described in Methods. The following variables were included as independent determinants: age, gender, type of permanent residence (colony or not), self-reported ethnicity, education, economic activity, financial status, marital status, number of persons in the family, and 2 measures of social support.

Pathological distress (total score of GHQ-12) decreased by better financial status compared to that of very bad (bad: B = -5.60, *p* < 0.001; fair: B = -6.86, p < 0.001; good: B = -7.90, p < 0.001; very good: B = -7.28, *p* < 0.001), higher perceived social support estimated by OSSS (B = -0.31, *p* < 0.001); and higher support in personal matters (B = -1.6, *p* = 0.042). Distress was increased by age (B = 0.06, *p* < 0.001); living in a colony (B = 0.98, *p* = 0.05), and increasing number of persons in the household (B = 0.18; *p* = 0.015). Educational attainment (ref. primary; secondary: 0.42, *p* = 0.045). Neither employment nor ethnicity proved to be significant determinants of pathological distress.

Higher WBI was predicted by not living in a colony (B = -0.95, *p* = 0.005), younger age (B = -0.086, *p* < 0.001), better financial status (ref: very bad; bad: B = 3.22, fair: B = 4.57, good: B = 6.46, very good: B = 5.79, for all: *p* < 0.001), fewer number of persons in the household (B = -1.86, *p* = 0.014), and increased level of perceived social support (B = 0.382, *p* < 0.001).

Life satisfaction (LS) was also positively determined by younger age (B = -0.028, *p* < 0.001), better than very bad financial status (bad: B = 1.61, fair: B = 2.61, good: B = 3.14, very good: B = 3.03, for all: p < 0.001), higher perceived social support (B = 0.14, *p* < 0.001), and lower number of persons in the household (B = -0.075, *p* = 0.024). Interestingly, having primary education compared to less than primary decreased life satisfaction (B = -0.34, *p* = 0.020). Type of residence or ethnicity were removed from the model as nonsignificant determinants of pathological distress.

### 3.4. Modeling the principal component of mental health

As described in Methods, all three mental status variables (WBI, LS, GHQ-12) were strongly correlated so principal component analysis was performed that yielded one factor, Component1 (Comp1, explaining 74% of the total variance) that was defined as the dependent variable. To estimate the effect of determinant variables on Comp1, generalized linear modeling (glm) was used by backward stepwise estimation removing variables at alpha = 0.1 as described in Methods. Variables found to be independent determinants of Component1 are shown in Table 4. Recalling that increasing values of the dependent variable (Component1) reflect worse mental health, one can conclude that living in a colony (segregated settlement), being older, and not being employed because of specific reasons significantly increases the score of Comp1 reflecting worsening mental health. In opposition, not being in the worst (very bad) financial status and having social support as assessed by OSSS and support in personal matters significantly decreases the score of Comp1, that is, improves mental health.

**Table 4:** Determinants of the principal component (Comp1) of mental health.

Independent variables	B	SE	*p* value	95% Confidence Interval	
				Lower bound	Upper bound
Type of permanent residence is not colony (ref: colony)	0.26	0.07	<0.001	0.128	0.396
Age (years)	0.01	0.00	0.002	0.003	0.013
Economic activity (ref: employed)					
inactive	0.16	0.08	0.046	0.003	0.322
Financial status (ref: very bad)					
bad	−1.15	0.18	<0.001	−1.509	−0.794
fair	−1.41	0.17	<0.001	−1.750	−1.074
good	−1.59	0.19	<0.001	−1.949	−1.223
very good	−1.51	0.25	<0.001	−1.996	−1.022
Social support (OSSS-3)	−0.067	0.02	<0.001	−0.101	−0.032
Support in personal matters (ref: no)					
yes	−0.37	0.16	0.024	−0.683	−0.049

OSSS-3: Oslo Social Support Scale.

## 4. Discussion

The main objective of this study was to investigate differences in mental health characteristics (subjective well-being, life satisfaction, and pathological distress) and social support between the general adult population and adult residents of segregated settlements (colonies) as well as between self-identified Roma and non-Roma in Hungary. Both divisions of the samples (by type of settlement and by self-identified ethnicity) into subgroups had shown that the subgroups of colony dwellers as well as self-identified Roma had significantly higher proportions of women; were younger, had lower educational levels, lower economic activity, worse financial status, and larger families (cohabiting persons) than those not living in colonies, and not identifying as Roma, respectively. There were strongly significant unfavorable differences in all 3 measures of mental health at the expense of colony dwellers and self-identified Roma as opposed to non-colony dwellers and non-Roma. We have also shown that residing in colonies does not necessarily equal being Roma since 32.2% of colony-dwellers self-identified themselves as Hungarian. Parsing out the contribution of ethnic identity and being a colony dweller – among other sociodemographic determinants – to mental health resulted in the conclusion that ethnic identity had no contribution to mental health when segregated housing conditions, age, educational attainment, employment, financial status, and social support are also taken into consideration. Self-reported ethnicity was not an independent variable of GHQ, WBI or LS when investigated separately, and ethnicity was also removed from the model when determinants of Component1 were modeled.

Our results are consistent with other comparative studies that have found that well-being and life satisfaction are unfavorable among Roma (15) when investigated in descriptive analyses, and that mental disorders such as functional disorders, depression, neurotic difficulties, and suicide are more common among them than in the general population (12, 13). The study by Kamberi et al. conducted on a non-representative sample of 8,399 Roma and 3,598 non-Roma adults from Central and Southeastern Europe and adjusted for sociodemographic variables found that significantly lower levels of life satisfaction and happiness among Roma compared to the majority population were not due to Roma identity in itself, but entirely to factors such as poorer health, lower income, lower education, poorer housing quality, stigmatisation in ethnic identity and discrimination (15). Other studies have also shown that immigrants in general consistently score lower on measures of life satisfaction and happiness than members of the majority population because of their lower material resources, lower social and human capital, and greater perceived discrimination (32, 33).

Universal, indicated, and selective prevention strategies can all be effective for the improvement of mental well-being and prevention of mental disorders and their risk conditions (34). However, our findings highlight the importance of taking into account fundamental socioeconomic determinants of health such as type of permanent residence especially whether it is a segregated settlement or not, and financial situation/income. All these should addressed when health promotion strategies are developed and structural level decisions are planned. Policies to eliminate segregated housing conditions, and policies to help disadvantaged pupils attain the highest possible education as a condition for decent employment could reduce health inequalities stemming from these unequal conditions of life (35–37). The new strategic EU framework for the equality, inclusion and participation of Roma does call for the reduction of housing deprivation (though reduction of segregation is not specified) and equal access to quality inclusive mainstream education (38), and these policy aims are also included in the Hungarian national strategy for inequality reduction.

We found similar levels of perceived social support in the observed populations despite significant differences in mental health. High level of social support is a strength of Roma communities and a promising point of health promotion interventions as has been shown by various projects employing Roma health mediators who live and work in Roma communities bridging the gap between professionals and community members, dispelling fear and mistrust, helping Roma to seek and appropriately use health services (39).

### 4.1. Strengths and limitations

This study is among only a handful of studies that collected data among inhabitants of segregated settlements (often designated as Roma settlements or Roma colonies) along with data on ethnic identity. Our samples are representative of two counties of Hungary in terms of type of residence (segregated vs. non-segregated settlements), age, and educational level. Sampling bias was minimized by random sampling and by employing interviewers who were familiar with the target populations. The latter also contributed to a high response rate. Standard questionnaires were used to measure variables that enabled reliable comparisons between the samples.

One of the limitations is the cross-sectional nature of the study that does not allow conclusions about cause-effect relationships, merely points to ones that should be further investigated. Our correlational analyses reveal only the association between social factors and the mental health characteristics of the sample, however, the direction of the relationships require further investigation. The heterogeneity of the two groups particularly in terms of financial status and education must also be noted. Exploring the pathways in which demographics, segregation, and mental health are related require more studies, in part focusing on causal pathways that is outside of the scope of our study. Follow-up studies focusing on the impact of improving well-known risk factors (eg. improving housing, increasing education) would also be of use.

The low internal consistency of the Oslo Social Support Scale imposes a further limitation on the interpretation of results regarding social support. Since Cronbach’s alpha is highly dependent on the number of items, short scales generally do not demonstrate high α values (40). The McDonald’s omega coefficient calculation gave a slightly higher, but still low internal reliability. This could be avoided in the future by using a more comprehensive instrument to assess perceived social support of such a deprived community as the segregated Roma.

### 4.2. Implications

Improving the mental health of Roma communities requires a fairly systematic view and implementation of interventions at multiple levels, including macro-level policies that address structural factors (housing, education, access to health care) and family and individual needs (41). Strategies to improve mental health at the population level include changes in legislation, policy making, and resource allocation. Legislative reforms have been shown to have a positive impact on mental health and correlate with improved access to care, diagnosis of mental illness, lower prevalence of poor mental health and suicide rates. Legislation benefiting mental health includes changes to mental health insurance coverage rules to improve financial protection (reduced financial burden) and expand access to mental health services (42).

Establishment of a dedicated body such as the Task Force on Community-Based Prevention Services in the United States could also contribute to policy decisions to improve mental health by making evidence-based recommendations for community-based prevention programs, services, and interventions –provided that it works as an independent, nongovernmental, nonpaid panel of public health and prevention experts (42). Recommendations should also be aimed at strengthening community-based prevention by creating mechanisms to increase ownership and social responsibility among community members. An example of such an initiative is the Communities that Care (CTC) program originating from the United States that has been adopted in the Netherlands and other countries (35).

## 5. Conclusion

The results of this study provide evidence on the notably disadvantaged mental health status of residents of segregated settlements compared to the general population in Hungary. The differences are measurable in lower levels of subjective well-being, lower life satisfaction and, most importantly, higher risk of mental morbidity compared to the majority population. The study also highlights the fact that housing segregation is not limited to Roma people; and strengthens the evidence base that housing conditions and financial status are major social determinants of mental health.

## Data availability statement

The raw data supporting the conclusions of this article will be made available by the authors, without undue reservation.

## Ethics statement

The studies involving human participants were reviewed and approved by Ethics Committee of the Hungarian Scientific Council on Health. The patients/participants provided their written informed consent to participate in this study.

## Author contributions

KK and ÉB: conceptualization, methodology, and project administration. KK and BO: formal analysis, data curation, and visualization. BO: writing—original draft preparation. KK and ÉB: writing—review and editing. KK: supervision and funding acquisition. All authors contributed to the article and approved the submitted version.

## Funding

The project was co-financed by the European Union under European Regional Development Fund (GINOP-2.3.2-15-2016-00005), as well as by the Hungarian Academy of Sciences (MTA11010 and TK2016-78). The preparation of the manuscript was funded by the EFOP-3.6.1-16-2016-00022 “Debrecen Venture Catapult program” project (co-financed by the European Union and the European Social Fund) and the János Bolyai Research Scholarship of the Hungarian Academy of Sciences.

## Conflict of interest

The authors declare that the research was conducted in the absence of any commercial or financial relationships that could be construed as a potential conflict of interest.

## Publisher’s note

All claims expressed in this article are solely those of the authors and do not necessarily represent those of their affiliated organizations, or those of the publisher, the editors and the reviewers. Any product that may be evaluated in this article, or claim that may be made by its manufacturer, is not guaranteed or endorsed by the publisher.
